# Branched-Chain Amino Acids, Alanine, and Thyroid Function: A Cross-Sectional, Nuclear Magnetic Resonance (NMR)-Based Approach from ELSA-Brasil

**DOI:** 10.3390/metabo14080437

**Published:** 2024-08-06

**Authors:** Carolina Castro Porto Silva Janovsky, Vandrize Meneghini, William Tebar, Joao Roberto Maciel Martins, José Augusto Sgarbi, Patrícia de Fatima dos Santos Teixeira, Steven R. Jones, Michael J. Blaha, Peter P. Toth, Paulo A. Lotufo, Marcio S. Bittencourt, Raul D. Santos, Itamar S. Santos, Layal Chaker, Isabela M. Bensenor

**Affiliations:** 1 Center for Clinical and Epidemiological Research, University Hospital, University of São Paulo, São Paulo 05508-000, Brazil; vandrize@usp.br (V.M.); tebar@usp.br (W.T.); palotufo@usp.br (P.A.L.); itamarss@usp.br (I.S.S.); isabensenor@hu.usp.br (I.M.B.); 2 Division of Endocrinology, Federal University of São Paulo/Escola Paulista de Medicina, São Paulo 04039-032, Brazil; j.martins@unifesp.br; 3 Division of Endocrinology, Faculty of Medicine of Marília (FAMEMA), Marília 17519-030, Brazil; jasgarbi@famema.br; 4 Medicine School, Federal University of Rio de Janeiro, Rio de Janeiro 21044-020, Brazil; pfatima@hucff.ufrj.br; 5 Johns Hopkins, Ciccarone Center for the Prevention of Heart Disease, Baltimore, MD 21287, USA; sjones64@jhmi.edu (S.R.J.); mblaha1@jhmi.edu (M.J.B.); 6 CGH Medical Center, Department of Preventive Cardiology, Sterling, IL 61081, USA; peter.toth@cghmc.com; 7 Department of Medicine and Department of Radiology, University of Pittsburgh, Pittsburgh, PA 15213, USA; bittencourtms@upmc.edu; 8 Heart Institute (InCor), University of São Paulo, São Paulo 05403-900, Brazil; raul.santos@incor.usp.br; 9 Hospital Israelita Albert Einstein, São Paulo 05652-900, Brazil; 10 Department of Internal Medicine and Rotterdam Thyroid Center, Erasmus University Medical Center, P.O. Box 2040, 3000 CA Rotterdam, The Netherlands; l.chaker@erasmusmc.nl; 11 Department of Epidemiology, Erasmus University Medical Center, P.O. Box 2040, 3000 CA Rotterdam, The Netherlands

**Keywords:** BCAAs, amino acids, thyroid, hypothyroidism, NMR spectroscopy

## Abstract

The association of thyroid function with essential and non-essential amino acids is understudied, despite their common metabolic roles. Thus, our aim was to evaluate the association of thyroid function with the levels of branched-chain amino acids (BCAAs—leucine, isoleucine, and valine) and of alanine in the general population. We utilized data from the São Paulo research center of ELSA-Brasil, a longitudinal population-based cohort study. Thyroid parameters included thyroid stimulating hormone (TSH), free T4 and free T3 levels, and the FT4:FT3 ratio. BCAAs and alanine were analyzed on a fully automated NMR platform. The current analysis included euthyroid participants and participants with subclinical hyperthyroidism and hypothyroidism. We used Pearson’s coefficient to quantify the correlation between thyroid-related parameters and amino acids. Linear regression models were performed to analyze whether thyroid parameters were associated with BCAAs and alanine levels. We included 4098 participants (51.3 ± 9.0 years old, 51.5% women) in this study. In the most adjusted model, higher levels of TSH were associated with higher levels of alanine, FT4 levels were inversely associated with isoleucine levels, FT3 levels were statistically significant and positively associated with valine and leucine, and the T3:T4 ratio was positively associated with all amino acids. We observed that subclinical hypothyroidism was positively associated with isoleucine and alanine levels in all models, even after full adjustment. Our findings highlight the association of subclinical hypothyroidism and thyroid-related parameters (including TSH, free T4, free T3, and FT4:FT3 ratio) with BCAAs and alanine. Further studies are needed to explore the mechanisms underlying this association. These insights contribute to our understanding of the influence of thyroid-related parameters on BCAA and alanine metabolism.

## 1. Introduction

Thyroid dysfunction plays a crucial role in elevating cardiometabolic risk through its profound impact on metabolic processes [1,2]. Thyroid hormones, primarily thyroxine (T4) and triiodothyronine (T3), regulate the basal metabolic rate, lipid metabolism, and glucose homeostasis [3]. Amino acid metabolism also seems to be impaired by thyroid dysfunctions; thus, branched-chain amino acids, including leucine, isoleucine, and valine, and also alanine, could be interpreted as a potential risk biomarker for cardiometabolic disease [4,5,6,7,8].

Branched-chain amino acids (BCAAs) are a group of essential amino acids that are not synthetized in animals and must be obtained through diet [9]. Evidence has accumulated recently that BCAAs are not only required for protein synthesis and nutrition but may also have a critical role in intracellular metabolism, with consequences for insulin resistance and mitochondrial dysfunction [4,5,6,7,8,9,10,11]. Also, it has been demonstrated that BCAAs and other amino acids (tryptophan and lysine) are markers of non-alcoholic steatohepatitis [12]. A previous study from our group has demonstrated that higher levels of BCAAs are associated with a worse cardiometabolic profile and with an increased incidence of type 2 diabetes after 4 years [13].

Alanine, on the other hand, is a non-essential amino acid that plays a crucial role in various biological processes [14,15,16], especially in the metabolic pathway through the glucose–alanine cycle (gluconeogenesis) [16,17,18,19]. Approximately 60% of alanine is derived from BCAAs [20], so its levels are intimately associated with BCAA metabolism. 

Disruptions in thyroid function can lead to imbalances in BCAAs and alanine, which in turn exacerbate metabolic derangements, contributing to cardiovascular diseases [21,22,23,24]. In hypothyroidism, there is a downregulation of essential amino acid oxidation, especially of leucine [24,25]. On the other hand, in hyperthyroidism, high levels of T3 increase BCAA oxidation and positively regulate mechanistic target of rapamycin (mTOR) [26,27], increasing thermogenesis [28,29]. There is also some evidence to suggest that BCAAs may alter the levels of thyroid hormones in animal models, but this is not fully elucidated [30,31]. In human research, only a positive correlation between thyroid hormone (T4) and the levels of BCAAs has been observed [32].

Thyroid dysfunctions and BCAAs are pivotal players in the complex interplay of metabolic processes that influence cardiovascular health. However, the associations between subclinical thyroid disease and essential and non-essential amino acids are not well investigated. Thus, we aimed to investigate the association of TSH, free T4 and free T3 levels, and the FT4:FT3 ratio with BCAAs and alanine levels in ELSA-Brasil participants with both subclinical thyroid diseases and with normal thyroid function.

## 2. Materials and Methods

### 2.1. Participants

**Figure 1:**
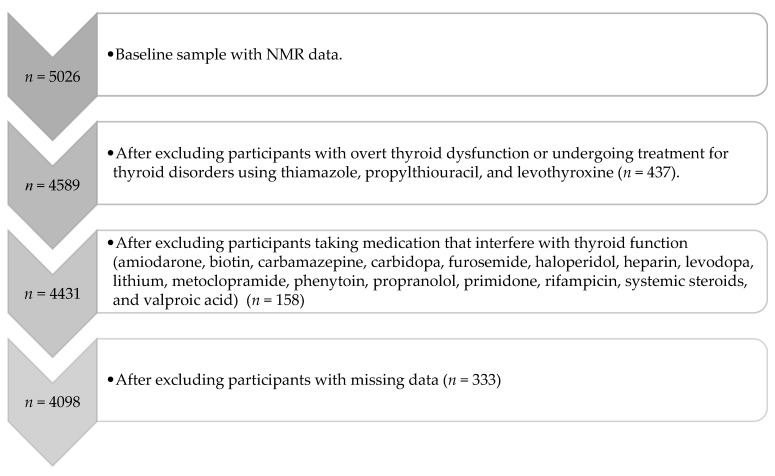
Flowchart of participants.

The ELSA-Brasil is a large, multicenter and prospective cohort study that has been following a population of middle-aged and older Brazilian adults for 15 years. The baseline data collection occurred between 2008 and 2010 with a widely comprehensive health assessment, which included laboratory and imaging tests. The design and detailed information about ELSA-Brasil have been published elsewhere [33,34,35,36]. In the current analysis, we included the participants from the São Paulo research center of ELSA-Brasil [35] with nuclear magnetic resonance (NMR) data available at baseline (*n* = 5026). Then, we excluded (1) those with overt thyroid dysfunction or using medication to treat thyroid diseases (thiamazole, propylthiouracil, and levothyroxine)—*n* = 437; (2) those that were taking medication that interfere with thyroid function (amiodarone, biotin, carbamazepine, carbidopa, furosemide, haloperidol, heparin, levodopa, lithium, metoclopramide, phenytoin, propranolol, primidone, rifampicin, systemic steroids, and valproic acid)—*n* = 158; and (3) those with missing data in any independent variable or covariate—*n* = 333. The total number of participants analyzed was 4098 and it included those with subclinical thyroid diseases and with normal thyroid function (Figure 1). The institutional ethics committee approved this study, and written informed consent was obtained from all participants.

### 2.2. Thyroid-Related Parameters

Venous blood samples were drawn in the morning after a 12 h overnight fast (6.30 a.m. to 9.00 a.m.). TSH (normal range: 0.40–4.00 mIU/L), FT4 (0.93–1.70 ng/dL), and FT3 (0.20–0.44 ng/dL) were determined by a third-generation immunoenzymatic assay (Roche Diagnostic, Mannheim, Germany). The analysis included euthyroid participants (TSH levels from 0.40 to 4.00 mIU/L with no history of levothyroxine or anti-thyroid drugs use), participants with subclinical hyperthyroidism (TSH levels lower than 0.40 mIU/L and FT4 levels between 0.93 and 1.70 ng/dL), and subclinical hypothyroidism (TSH > 4.00 mIU/L with FT4 0.93 to 1.70 ng/dL).

### 2.3. Amino Acids Evaluation

Blood was drawn into lavender-top EDTA collection tubes. Blood samples were promptly centrifuged (at 3000 rpm for 10 to 15 min at room temperature) and the separated plasma was refrigerated immediately. Refrigerated plasma specimens may be stored for up to 7 days without affecting NMR results [37].

Aliquots were frozen at −80 °C for further determinations of BCAAs (valine, leucine, isoleucine) and alanine, performed by proton nuclear magnetic resonance (1H NMR) spectroscopy. NMR LipoProfile analyses of fasting EDTA plasma samples using the NMR Profiler platform at LipoScience (now Labcorp, Morrisville, NC, USA) with the LP4 algorithm were performed [38]. The methyl signals from the three BCAAs and alanine in the 1H NMR spectrum produce distinct patterns which can then be used for quantification of isoleucine, leucine, and valine, as well as alanine. 

### 2.4. Other Baseline Variables

Body mass index (BMI) and waist circumference were measured using standard techniques.

Dyslipidemia was defined as a low-density lipoprotein (LDL) cholesterol level ≥130 mg/dL or the use of lipid- lowering medication. Total cholesterol, high-density lipoprotein (HDL) cholesterol, and triglycerides (glycerol phosphate peroxidase) were measured by enzymatic colorimetric assay (Siemens, Deerfield, MA, USA); LDL cholesterol was calculated using the Friedewald equation, except for cases with triglycerides >400 mg/dL, when an enzymatic colorimetric assay (ADVIA 1200, Siemens, Deerfield, MA, USA) was used.

Questionnaires addressed age, educational attainment (less than high school, high school and some college, complete college or higher); average monthly net family income in USD (<1245, 1245–3319, ≥3320) (at baseline, USD 1 = BRL 2); self-reported race (white, mixed, black, Asian, and indigenous); private health insurance plan (yes/no); and smoking and alcohol status (never, past, or current). Physical activity at leisure was assessed by the International Physical Activity Questionnaire and categorized as inactive, insufficiently active, and active [39,40]. All participants reported medication use in the two weeks prior to the interview.

### 2.5. Data Analyses

Data were presented as counts (*n*) and percentages (%) or means and standard deviations (SDs). Because the distribution of thyroid hormone levels and ratios were skewed and had excess kurtosis, a natural log transformation (ln) was performed (ln [original value + 1]). The comparison of the amino acids’ levels (leucine, isoleucine, valine, and alanine) according to the presence of subclinical hypothyroidism (reference category: euthyroidism) was performed using independent samples t tests and Levene’s test for equality variances. Univariate and multivariate Generalized Linear Models were performed to analyze whether the thyroid parameters and subclinical hypothyroidism were associated with the amino acid levels. Beta (β) and 95% confidence interval (95%CI) were presented for the following models: Model 1: crude analysis; Model 2: adjusted for sociodemographic variables (age, race, sex, educational level); and Model 3: adjusted for sociodemographic variables and health conditions (BMI, physical activity, diabetes, and smoking). We also performed a fourth model adding a quadratic term of each log-transformed thyroid function-related value (TSH, FT4, FT3, and the T3:T4 ratio) to verify whether there were non-linear associations. The Bayesian information criteria and likelihood ratio were analyzed. Sensitivity analyses were performed stratifying the participants by sex (female/male), age (<50/≥50 years old), presence of diabetes, and by restricting for euthyroid individuals. All the analyses were carried out using IBM SPSS v.26 software, adopting a significance level of 5% (*p* < 0.05). 

## 3. Results

We included 4098 participants (51.3 ± 9.0 years old, 51.5% women, BMI of 27.3 ± 4.9) in this study (Figure 1). Subclinical hypothyroidism was prevalent in 9.3% and subclinical hyperthyroidism in 0.6% of the study population. The demographic data of participants according to thyroid function are described in Table 1. Only self-reported race presented a statistically significant difference between euthyroid and subclinical hypothyroidism participants (*p* = 0.014).

**Figure 2:**
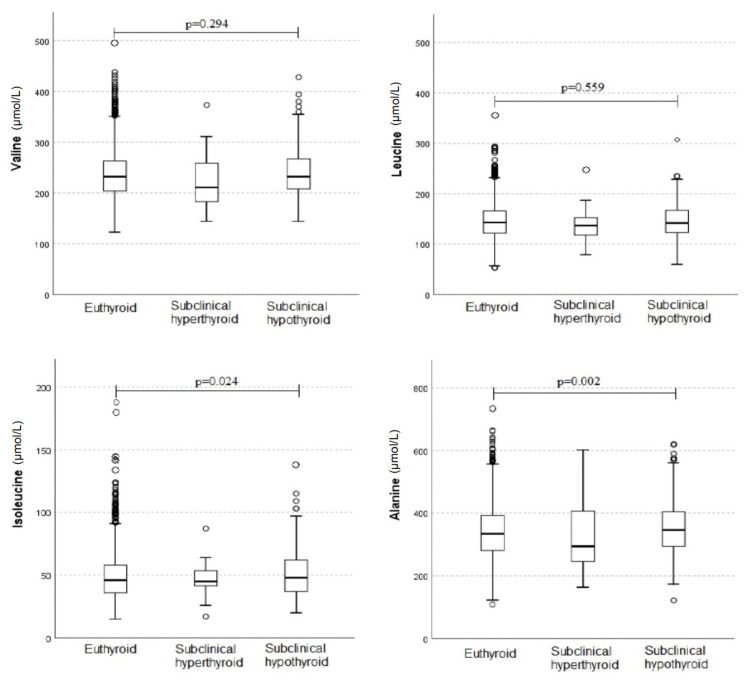
Comparison of valine, leucine, isoleucine, and alanine level distribution according to thyroid function. When compared to euthyroid participants, participants with subclinical hypothyroidism showed higher mean values of isoleucine (50.7 vs. 48.6; *p* = 0.024) and alanine (353.1 vs. 339.5; *p* = 0.002). All other comparisons were not significant (*p* > 0.05).

Figure 2 shows the distribution of valine, leucine, isoleucine, and alanine levels according to thyroid function. When compared to euthyroid participants, participants with subclinical hypothyroidism showed higher mean values of isoleucine (50.7 vs. 48.6; *p* = 0.024) and alanine (353.1 vs. 339.5; *p* = 0.002). All other comparison were not significant (*p* > 0.05).

Univariate and multivariate linear regression models were applied to analyze the association between thyroid parameters and amino acids in all participants (Table 2). Log-transformed levels of TSH, FT3, and the T3:T4 ratio were positively associated with all amino acids in the univariate analysis, while FT4 levels were only associated with leucine. After the adjustment for sociodemographic factors and health conditions, TSH remained associated with alanine levels (β: 14.44, 95% CI: 7.89; 20.99, *p* < 0.001). Log-transformed FT4 levels became negatively associated with isoleucine levels (β: −8.75, 95% CI: −15.05; −2.45, *p* = 0.006). Log-transformed FT3 levels remained positively associated with valine (β: 61.75, 95% CI: 25.87; 97.64, *p* < 0.001) and leucine (ꞵ: 43.39, 95% CI: 16.05; 70.73, *p* = 0.002). Finally, the T3:T4 ratio was positively associated with all amino acids in the final model (β range from 33.55 to 104.32; all *p* < 0.01). 

Adding a quadratic term to the models did not show consistent associations, nor did it improve overall model performance. Although significant associations between quadratic lnFT3 and lnT3:T4 appeared in some models, we detected that this result was due to an influential point with outlier T3 values. When this observation was excluded, the significant associations with quadratic terms vanished while the linear models did not change significantly.

Subclinical hypothyroidism was associated with higher levels of isoleucine and alanine in all models, even after full adjustment (isoleucine (β: 1.97, 95% CI: 0.36; 3.57, *p* = 0.017) and alanine (β: 10.18, 95% CI: 2.05; 18.30, *p* = 0.014)) (Figure S1—Supplementary Materials). The number of cases of subclinical hyperthyroidism was too small to be analyzed.

In the sensitivity analyses (Supplementary Tables S1–S4), we observed that log-transformed TSH levels remained positively associated with alanine in both sex and age groups, in those without diabetes, and in euthyroid individuals. The negative association between log-transformed FT4 levels and isoleucine was statistically significant in women, the younger group, those without diabetes, and euthyroid participants. FT4 levels were also negatively associated with valine in the younger group and in those without diabetes and with alanine levels in those without diabetes. Conversely, participants with diabetes showed a positive association between FT4 levels and those of valine, leucine, and isoleucine. There were positive associations between log-transformed FT3 levels and valine and leucine levels in women, the younger group, and in euthyroid individuals. FT3 values were also positively associated with valine in men and older adults and with all amino acids in participants without diabetes. The log-transformed T3:T4 ratio lost some associations after stratifying the analyses; however, we observed consistent associations with most of the amino acids in both sex and age groups, participants without diabetes, and euthyroid individuals. Subclinical hypothyroidism remained positively associated with alanine in the younger group and those without diabetes and with isoleucine only in older adults.

## 4. Discussion

To our knowledge, this is the largest study to address potential associations between subclinical thyroid dysfunction and thyroid-related parameters with BCAAs. In this study, we found associations between thyroid-related parameters and branched-chain amino acids and alanine. In the main analysis, TSH levels was positively associated with alanine. Free T3 levels were positively associated with valine and leucine levels, while free T4 levels was only negatively associated with isoleucine levels. Higher levels of the conversion ratio of FT4 to FT3 had a marked association with all analyzed amino acids (valine, leucine, isoleucine, and alanine). Younger age, the female sex, and the absence of diabetes were important determinants in the relationship of thyroid parameters and amino acids after sensitivity analyses.

A strict homeostasis between protein consumption and protein synthesis is needed to maintain balance in the tissue pools of BCAAs, as the principal source of these nutrients is the diet. Low levels of BCAAs have been correlated with malnutrition and increased mortality in cardiovascular disease [41,42], while excess BCAAs have been correlated with increased risk of insulin resistance, diabetes mellitus, and cardiovascular disease [5,6,8,13,21,43]. Thyroid hormones increase BCCA oxidation before altering energetic expenditure, glucose, and lipid metabolism [44,45,46]. 

Most of the studies have evaluated associations with leucine levels. Van der Boom et al. have shown that, in patients thyroidectomized for thyroid cancer, mild hyperthyroidism accelerates BCAA oxidation, increasing leucine flux, while hypothyroidism impairs endogenous rates of leucine appearance, its oxidation, and its non-oxidative disposal [24]. Thus, thyroid status directly regulates protein metabolism by reducing protein breakdown in hypothyroidism and increasing it in hyperthyroidism [47]. It is also known that leucine regulates mTOR, which is one of the main regulators of thermogenesis [7,21,29]. T3 also has direct effects on mTOR phosphorylation in brown adipose tissue (BAT) and is needed for the activation of thermogenesis in BAT [48], as well as for the browning of white adipose tissue [49,50,51]. Hyperthyroidism reduces intracellular BAT levels of leucine and arginine by stimulating oxidation [29] and leading to mitochondrial autophagy, activity, and turnover in BAT, which are essential for thermogenesis [29,52]. Our study shows that higher FT3 levels and T3:T4 ratio are correlated with higher leucine and valine levels, confirming such a relationship, which would probably lead to thermogenesis. However, isoleucine showed an opposite direction in our study, with a negative association with FT4. This could be due to the usual opposite behavior between serum T4 and T3 in disease status [53,54].

Regarding alanine, it is a non-essential amino acid that plays a crucial role in protein synthesis and energy metabolism and is intimately linked to leucine levels. The secretion of alanine from skeletal muscle depends on the amination of pyruvate, which occurs with 20% of the nitrogen coming from leucine [9]. Alanine also serves as a precursor for hepatic gluconeogenesis [9,17,19,55]. However, previous studies have shown contradictory results with alanine; it is associated with both hypo- and hyperthyroidism [44,45,46,56]. Our study revealed associations between TSH, T3:T4 ratio, subclinical hypothyroidism, and alanine levels. The positive association between TSH, the T3:T4 ratio, and alanine levels may reflect complex interactions between thyroid function, metabolic processes, and the body’s response to stress or illness.

Indeed, our study is the first to evaluate the T3:T4 ratio and its association with BCCAs and alanine. The T3:T4 ratio is used to evaluate the rate of T4 to T3 conversion, reflecting the peripheral sensitivity of thyroid hormones. An increase in the T3:T4 ratio represents a consequence of an adaptation to adverse metabolic conditions, which enhances the activity of type 2 deiodinase (DIO2) [57,58]. It could be considered an early marker of thyroid function’s effects on metabolic parameters such as obesity or non-alcoholic fatty liver disease [59] and even cardiovascular mortality prediction [60]. The association of this ratio with all amino acids analyzed substantiate the role of the conversion ratio as an early marker of thyroid impairment possibly affecting metabolic parameters [54,55].

This study has some strengths and limitations. The well-designed cohort, which was followed up for 15 years with uniform collection, storage, and analyses of serum samples using a highly reproducible method such as NMR spectroscopy, denotes the main strength of our study. This study is also innovative and could shed light into the role of thyroid dysfunction in overall metabolic health in future investigations because, as a cross-sectional analysis, we cannot establish causality. The small number of individuals with subclinical hyperthyroidism did not enable us to focus on this particular group of participants. Nevertheless, by using TSH, FT4, and FT3 as continuous variables, we were able to gain insight to the association of thyroid function in general with BCAAs and alanine. Even though ELSA-Brasil includes a sample with higher education and net family income compared to the general Brazilian population, this study presents a high social and ethnic diversity that is similar to the heterogeneous populations living in the metropolitan areas in Brazil. This implies that our external validity may extend to urban centers of similar characteristics both within and outside Brazil. Particularly in this analysis, we cannot know if a high educational level could influence nutrition and therefore serum amino acid levels because there are no data available about it in the general Brazilian population, which has, on average, a lower educational level and lower income. In addition, there are several similarities in the prevalence of behavioral risk factors and chronic conditions selected for assessment with similar procedures in ELSA-Brasil and in VIGITEL, an annually performed telephone-based behavioral risk factor survey producing representative data for adults living in Brazil’s 27 state capitals and Federal District [61].

## 5. Conclusions

In conclusion, our findings highlight the association between thyroid-related parameters, including thyroid hormones, TSH, and T3:T4 ratio and BCAA and alanine levels.

It was demonstrated that, after adjustment for demographic factors and comorbidities, low thyroid function, represented by high levels of TSH, low levels of FT4, high serum levels of FT3, and a higher T3:T4 ratio, was associated with higher levels of BCAAs and alanine, which could serve as a biomarker for higher cardiometabolic risk and insulin resistance. Further prospective studies are needed to explore the causality and the mechanisms underlying this association.

## Figures and Tables

**Table 1:** Descriptive characteristics of the participants according to thyroid function.

	All (*n* = 4098)	Thyroid Function		
		Euthyroid (*n* = 3693)	Subclinical Hyper (*n* = 23)	Subclinical Hypo (*n* = 382)
	*n* (%)	*n* (%)	*n* (%)	*n* (%)
Gender				
Men	1988 (48.5%)	1796 (48.6%)	7 (30.4%)	185 (48.4%)
Women	2110 (51.5%)	1897 (51.4%)	16 (69.6%)	197 (51.6%)
Education				
Up to some college	2316 (56.5%)	2091 (56.6%)	12 (52.2%)	213 (55.8%)
Completed college or more	1782 (43.5%)	1602 (43.4%)	11 (47.8%)	169 (44.2%)
Self-reported race ^a^				
Non-white	1725 (42.1%)	1574 (42.6%)	13 (56.5%)	138 (36.1%)
White	2373 (57.9%)	2119 (57.4%)	10(43.5%)	244 (63.9%)
Physical activity				
Inactive	2703 (66.0%)	2430 (65.8%)	13 (56.5%)	260 (68.1%)
Insufficiently active	459 (11.2%)	412 (11.2%)	2 (8.7%)	45 (11.8%)
Active	936 (22.8%)	851 (23.0%)	8 (34.8%)	77 (20.2%)
Smoking				
Never smoked	2170 (53.0%)	1940 (52.5%)	11 (47.8%)	219 (57.3%)
Past or current smoker	1928 (47.0%)	1753 (47.5%)	12 (52.2%)	163 (42.7%)
Diabetes mellitus				
No	3384 (82.6%)	3041 (82.3%)	19 (82.6%)	324 (84.8%)
Yes	714 (17.4%)	652 (17.7%)	4 (17.4%)	58 (15.2%)

^a^ Statistically significant difference between euthyroid and subclinical hypothyroidism participants.

**Table 2:** Crude and adjusted linear association between thyroid hormones and amino acids to all participants.

	Valine		Leucine		Isoleucine		Alanine	
	β (CI 95%)	*p*	β (CI 95%)	*p*	β (CI 95%)	*p*	β (CI 95%)	*p*
Model 1—Crude								
TSH ^a^	4.31 (0.56; 8.07)	0.024	2.96 (0.26; 5.67)	0.032	1.87 (0.38; 3.36)	0.014	19.46 (12.60; 26.32)	<0.001
FT4 ^a^	13.04 (−5.08; 31.16)	0.158	16.09 (3.04; 29.14)	0.016	1.02 (−6.17; 8.21)	0.781	−16.31 (−49.53; 16.91)	0.336
FT3 ^a^	243.27 (201.49; 285.04)	<0.001	170.53 (140.40; 200.66)	<0.001	76.35 (59.68; 93.01)	<0.001	148.73 (71.03; 226.39)	<0.001
T3:T4 ratio ^a^	158.34 (119.96; 196.72)	<0.001	98.31 (70.59; 126.02)	<0.001	55.61 (40.37; 70.86)	<0.001	148.60 (77.83; 219.37)	<0.001
Model 2—Adjusted for sociodemographic variables (age, race, sex, educational level)								
TSH ^a^	2.89 (−0.47; 6.25)	0.092	2.41 (−0.06; 4.87)	0.055	1.53 (0.17; 2.89)	0.028	15.89 (9.16; 22.62)	<0.001
FT4 ^a^	−30.81 (−47.11; −14.50)	<0.001	−11.87 (−23.83; 0.10)	0.052	−14.45 (−21.05; −7.85)	<0.001	−51.25 (−84.00; −18.50)	0.002
FT3 ^a^	79.07 (39.61; 118.54)	<0.001	52.88 (23.95; 81.81)	<0.001	11.84 (−4.17; 27.84)	0.147	82.01 (3.69; 162.34)	0.040
T3:T4 ratio ^a^	111.54 (76.91; 146.18)	<0.001	59.04 (33.60; 84.48)	<0.001	35.14 (21.09; 49.19)	<0.001	157.26 (87.56; 226.95)	<0.001
Model 3—Adjusted for model 2 + health conditions (BMI, physical activity, diabetes, and smoking)								
TSH ^a^	1.41 (−1.65; 4.47)	0.367	2.00 (−0.34; 4.33)	0.094	1.10 (−0.19; 2.40)	0.095	14.44 (7.89; 20.99)	<0.001
FT4 ^a^	−11.30 (−26.23; 3.63)	0.138	−2.00 (−13.38; 9.37)	0.730	−8.75 (−15.05; −2.45)	0.006	−7.72 (−39.72; 28.29)	0.637
FT3 ^a^	61.75 (25.87; 97.64)	0.001	43.39 (16.05; 70.73)	0.002	6.58 (−8.59; 21.75)	0.396	64.98 (−11.99; 141.95)	0.098
T3:T4 ratio ^a^	62.17 (30.20; 94.13)	<0.001	33.55 (9.18; 57.92)	0.007	20.46 (6.95; 33.97)	0.003	104.32 (35.78; 172.86)	0.003

Note: **^a^** log-transformed variables. β represents the slope coefficient of the regression line in sensitivity analyses.

## Data Availability

The raw data supporting the conclusions of this article will be made available by the authors on request due to ethical reasons and proceedings of the study.

## Supplementary Materials

The following supporting information can be downloaded at: https://www.mdpi.com/article/10.3390/metabo14080437/s1, Figure S1: Forest plots showing crude and adjusted linear association between subclinical hypothyroidism and amino acids; Table S1: Crude and adjusted linear association between thyroid parameters, subclinical hypothyroidism, and amino acids according to sex; Table S2: Crude and adjusted linear association between thyroid parameters, subclinical hypothyroidism, and amino acids according to age group; Table S3: Crude and adjusted linear association between thyroid parameters, subclinical hypothyroidism, and amino acids according to the presence of diabetes; Table S4: Crude and adjusted linear association between thyroid parameters and amino acids for euthyroid participants exclusively.

## References

[1] Yamakawa H., Kato T.S., Noh J.Y., Yuasa S., Kawamura A., Fukuda K., Aizawa Y. (2021). Thyroid hormone plays an important role in cardiac function: From bench to bedside. Front. Physiol..

[2] Neves J.S., Fontes-Carvalho R., Borges-Canha M., Leite A.R., von Hafe M., Vale C., Martins S., Guimarães J.T., Carvalho D., Leite-Moreira A. (2022). Association of thyroid function, within the euthyroid range, with cardiovascular risk: The epiporto study. Front. Endocrinol..

[3] Yavuz S., Salgado Nunez del Prado S., Celi F.S. (2019). Thyroid hormone action and energy expenditure. J. Endocr. Soc..

[4] Newgard C.B., An J., Bain J.R., Muehlbauer M.J., Stevens R.D., Lien L.F., Haqq A.M., Shah S.H., Arlotto M., Slentz C.A. (2009). A branched-chain amino acid-related metabolic signature that differentiates obese and lean humans and contributes to insulin resistance. Cell Metab..

[5] Connelly M.A., Wolak-Dinsmore J., Dullaart R.P.F. (2017). Branched chain amino acids are associated with insulin resistance independent of leptin and adiponectin in subjects with varying degrees of glucose tolerance. Metab. Syndr. Relat. Disord..

[6] Bandt J.-P.D., Coumoul X., Barouki R. (2022). Branched-chain amino acids and insulin resistance, from protein supply to diet-induced obesity. Nutrients.

[7] Holeček M. (2018). Branched-chain amino acids in health and disease: Metabolism, alterations in blood plasma, and as supplements. Nutr. Metab..

[8] Lynch C.J., Adams S.H. (2014). Branched-chain amino acids in metabolic signalling and insulin resistance. Nat. Rev. Endocrinol..

[9] Neinast M., Murashige D., Arany Z. (2018). Branched chain amino acids. Annu. Rev. Physiol..

[10] Batch B.C., Hyland K., Svetkey L.P. (2014). Branch chain amino acids. Curr. Opin. Clin. Nutr..

[11] Sperringer J.E., Addington A., Hutson S.M. (2017). Branched-chain amino acids and brain metabolism. Neurochem. Res..

[12] Goffredo M., Santoro N., Tricò D., Giannini C., D’Adamo E., Zhao H., Peng G., Yu X., Lam T.T., Pierpont B. (2017). A branched-chain amino acid-related metabolic signature characterizes obese adolescents with non-alcoholic fatty liver disease. Nutrients.

[13] de Almeida-Pititto B., Dualib P.M., Jordão M.C., Izar Helfenstein Fonseca M., Jones S.R., Blaha M.J., Toth P.P., Santos R.D., Bensenor I.M., Ferreira S.R.G. (2021). Branched-chain amino acids predict incident diabetes in the brazilian longitudinal study of adult health—Elsa-brasil. Diabetes Res. Clin. Pract..

[14] Schutz Y. (2011). Protein turnover, ureagenesis and gluconeogenesis. Int. J. Vitam. Nutr. Res..

[15] Odessey R., Khairallah E.A., Goldberg A.L. (1974). Origin and possible significance of alanine production by skeletal muscle. J. Biol. Chem..

[16] de Souza Galia W.B., Biazi G.R., Frasson-Uemura I.G., Miksza D.R., Zaia C.T.B.V., Zaia D.A.M., de Souza H.M., Bertolini G.L. (2021). Gluconeogenesis is reduced from alanine, lactate and pyruvate, but maintained from glycerol, in liver perfusion of rats with early and late sepsis. Cell Biochem. Funct..

[17] Felig P., Pozefsk T., Marlis E., Cahill G.F. (1970). Alanine: Key role in gluconeogenesis. Science.

[18] Adeva-Andany M., López-Ojén M., Funcasta-Calderón R., Ameneiros-Rodríguez E., Donapetry-García C., Vila-Altesor M., Rodríguez-Seijas J. (2014). Comprehensive review on lactate metabolism in human health. Mitochondrion.

[19] Adeva-Andany M.M., Pérez-Felpete N., Fernández-Fernández C., Donapetry-García C., Pazos-García C. (2016). Liver glucose metabolism in humans. Biosci. Rep..

[20] Haymond M.W., Miles J.M. (1982). Branched chain amino acids as a major source of alanine nitrogen in man. Diabetes.

[21] Batch B.C., Shah S.H., Newgard C.B., Turer C.B., Haynes C., Bain J.R., Muehlbauer M., Patel M.J., Stevens R.D., Appel L.J. (2013). Branched chain amino acids are novel biomarkers for discrimination of metabolic wellness. Metabolis.

[22] Sun L., Goh H.J., Verma S., Govindharajulu P., Sadananthan S.A., Michael N., Henry C.J., Goh J.P., Velan S.S., Leow M.K. (2022). Brown adipose tissues mediate the metabolism of branched chain amino acids during the transitioning from hyperthyroidism to euthyroidism (tribute). Sci. Rep..

[23] Giacco A., Cioffi F., Cuomo A., Simiele R., Senese R., Silvestri E., Amoresano A., Fontanarosa C., Petito G., Moreno M. (2022). Mild endurance exercise during fasting increases gastrocnemius muscle and prefrontal cortex thyroid hormone levels through differential bhb and bcaa-mediated bdnf-mtor signaling in rats. Nutrients.

[24] van der Boom T., Gruppen E.G., Lefrandt J.D., Connelly M.A., Links T.P., Dullaart R.P.F. (2020). Plasma branched chain amino acids are lower in short-term profound hypothyroidism and increase in response to thyroid hormone supplementation. Scand. J. Clin. Lab. Investig..

[25] Rochon C., Tauveron I., Dejax C., Benoit P., Capitan P., Bayle G., Prugnaud J., Fabricio A., Berry C., Champredon C. (2000). Response of leucine metabolism to hyperinsulinemia in hypothyroid patients before and after thyroxine replacement1. J. Clin. Endocrinol. Metab..

[26] Adegoke O.A.J., Abdullahi A., Tavajohi-Fini P. (2012). Mtorc1 and the regulation of skeletal muscle anabolism and mass. Appl. Physiol. Nutr. Metab..

[27] Mann G., Mora S., Madu G., Adegoke O.A.J. (2021). Branched-chain amino acids: Catabolism in skeletal muscle and implications for muscle and whole-body metabolism. Front. Physiol..

[28] Varela L., Martínez-Sánchez N., Gallego R., Vázquez M.J., Roa J., Gándara M., Schoenmakers E., Nogueiras R., Chatterjee K., Tena-Sempere M. (2012). Hypothalamic mtor pathway mediates thyroid hormone-induced hyperphagia in hyperthyroidism. J. Pathol..

[29] Yau W.W., Singh B.K., Lesmana R., Zhou J., Sinha R.A., Wong K.A., Wu Y., Bay B.H., Sugii S., Sun L. (2019). Thyroid hormone (t3) stimulates brown adipose tissue activation via mitochondrial biogenesis and mtor-mediated mitophagy. Autophagy.

[30] Carew L.B., Evarts K.G., Alster F.A. (1997). Growth and plasma thyroid hormone concentrations of chicks fed diets deficient in essential amino acids. Poult. Sci..

[31] Carew L.B., Evarts K.G., Alster F.A. (1998). Growth, feed intake, and plasma thyroid hormone levels in chicks fed dietary excesses of essential amino acids. Poult. Sci..

[32] Krishnamurthy H.K., Reddy S., Jayaraman V., Krishna K., Song Q., Rajasekaran K.E., Wang T., Bei K., Rajasekaran J.J. (2021). Effect of micronutrients on thyroid parameters. J. Thyroid Res..

[33] Aquino E.M., Barreto S.M., Bensenor I.M., Carvalho M.S., Chor D., Duncan B.B., Lotufo P.A., Mill J.G., Molina Mdel C., Mota E.L. (2012). Brazilian longitudinal study of adult health (elsa-brasil): Objectives and design. Am. J. Epidemiol..

[34] Schmidt M.I., Duncan B.B., Mill J.G., Lotufo P.A., Chor D., Barreto S.M., Aquino E.M., Passos V.M., Matos S.M., Molina Mdel C. (2015). Cohort profile: Longitudinal study of adult health (elsa-brasil). Int. J. Epidemiol..

[35] Pereira A.C., Bensenor I.M., Fedeli L.M., Castilhos C., Vidigal P.G., Maniero V., Leite C.M., Pimentel R.A., Duncan B.B., Mill J.G. (2012). Delineamento e implementação do biobanco do elsa-brasil: Estudo prospectivo na população brasileira. Rev. De Saúde Pública.

[36] Bensenor I.M., Griep R.H., Pinto K.A., de Faria C.P., Felisbino-Mendes M., Caetano E., Albuquerque L.S., Schmidt M.I. (2012). Rotinas de organização de exames e entrevistas no centro de investigação elsa-brasil. Rev. De Saúde Pública.

[37] Jeyarajah E.J., Cromwell W.C., Otvos J.D. (2006). Lipoprotein particle analysis by nuclear magnetic resonance spectroscopy. Clin. Lab. Med..

[38] Matyus S.P., Braun P.J., Wolak-Dinsmore J., Jeyarajah E.J., Shalaurova I., Xu Y., Warner S.M., Clement T.S., Connelly M.A., Fischer T.J. (2014). NMR measurement of LDL particle number using the vantera^®^ clinical analyzer. Clin. Biochem..

[39] Craig C.L., Marshall A.L., Sjöström M., Bauman A.E., Booth M.L., Ainsworth B.E., Pratt M., Ekelund U., Yngve A., Sallis J.F. (2003). International physical activity questionnaire; 12-country reliability and validity. Med. Sci. Sports Exerc..

[40] Lee P.H., Macfarlane D.J., Lam T., Stewart S.M. (2011). Validity of the international physical activity questionnaire short form (ipaq-sf): A systematic review. Int. J. Behav. Nutr. Phys. Act..

[41] Klobučar I., Vidović L., Arih I., Lechleitner M., Pregartner G., Berghold A., Habisch H., Madl T., Frank S., Degoricija V. (2023). Low valine serum levels predict increased 1-year mortality in acute heart failure patients. Biomolecules.

[42] Otvos J.D., Shalaurova I., May H.T., Muhlestein J.B., Wilkins J.T., McGarrah R.W., Kraus W.E. (2023). Multimarkers of metabolic malnutrition and inflammation and their association with mortality risk in cardiac catheterisation patients: A prospective, longitudinal, observational, cohort study. Lancet Healthy Longev..

[43] Wiklund P., Zhang X., Pekkala S., Autio R., Kong L., Yang Y., Keinänen-Kiukaanniemi S., Alen M., Cheng S. (2016). Insulin resistance is associated with altered amino acid metabolism and adipose tissue dysfunction in normoglycemic women. Sci. Rep..

[44] Riis A.L., Jørgensen J.O., Ivarsen P., Frystyk J., Weeke J., Møller N. (2008). Increased protein turnover and proteolysis is an early and primary feature of short-term experimental hyperthyroidism in healthy women. J. Clin. Endocrinol. Metab..

[45] Riis A.L., Jørgensen J.O., Gjedde S., Nørrelund H., Jurik A.G., Nair K.S., Ivarsen P., Weeke J., Møller N. (2005). Whole body and forearm substrate metabolism in hyperthyroidism: Evidence of increased basal muscle protein breakdown. Am. J. Physiol. Endocrinol. Metab..

[46] Kobayashi R., Shimomura Y., Otsuka M., Popov K.M., Harris R.A. (2000). Experimental hyperthyroidism causes inactivation of the branched-chain α-ketoacid dehydrogenase complex in rat liver. Arch. Biochem. Biophys..

[47] Morrison W.L., Gibson J.N.A., Jung R.T., Rennie M.J. (1988). Skeletal muscle and whole body protein turnover in thyroid disease. Eur. J. Clin. Investig..

[48] Christoffolete M.A., Linardi C.C., de Jesus L., Ebina K.N., Carvalho S.D., Ribeiro M.O., Rabelo R., Curcio C., Martins L., Kimura E.T. (2004). Mice with targeted disruption of the dio2 gene have cold-induced overexpression of the uncoupling protein 1 gene but fail to increase brown adipose tissue lipogenesis and adaptive thermogenesis. Diabetes.

[49] Senese R., Cioffi F., De Matteis R., Petito G., de Lange P., Silvestri E., Lombardi A., Moreno M., Goglia F., Lanni A. (2019). 3,5 Diiodo-l-thyronine (t2) promotes the browning of white adipose tissue in high-fat diet-induced overweight male rats housed at thermoneutrality. Cells.

[50] Bargut T.C.L., Souza-Mello V., Aguila M.B., Mandarim-De-Lacerda C.A. (2017). Browning of white adipose tissue: Lessons from experimental models. Horm. Mol. Biol. Clin. Investig..

[51] Cioffi F., Gentile A., Silvestri E., Goglia F., Lombardi A. (2018). Effect of iodothyronines on thermogenesis: Focus on brown adipose tissue. Front. Endocrinol..

[52] Steinhoff K.G., Krause K., Linder N., Rullmann M., Volke L., Gebhardt C., Busse H., Stumvoll M., Blüher M., Sabri O. (2021). Effects of hyperthyroidism on adipose tissue activity and distribution in adults. Thyroid.

[53] Salas-Lucia F., Bianco A.C. (2022). T3 levels and thyroid hormone signaling. Front. Endocrinol..

[54] Abbey E., Mcgready J., Simonsick E., Mammen J. (2020). T3:T4 ratio can distinguish between adaptive changes and true subclinical hypothyroidism in older adults. Innov. Aging.

[55] Berghe G. (1996). Disorders of gluconeogenesis. J. Inherit. Metab. Dis..

[56] Müller M.J., Seitz H.J. (1984). Thyroid hormone action on intermediary metabolism. Klin. Wochenschr..

[57] Gökmen F.Y., Ahbab S., Ataoğlu H.E., Türker B.Ç., Çetin F., Türker F., Mamaç R.Y., Yenigün M. (2016). Ft3/Ft4 ratio predicts non-alcoholic fatty liver disease independent of metabolic parameters in patients with euthyroidism and hypothyroidism. Clinics.

[58] Hoermann R., Midgley J.E.M., Larisch R., Dietrich J.W. (2015). Homeostatic control of the thyroid–pituitary axis: Perspectives for diagnosis and treatment. Front. Endocrinol..

[59] Park S.Y., Park S.E., Jung S.W., Jin H.S., Park I.B., Ahn S.V., Lee S. (2017). Free triiodothyronine/free thyroxine ratio rather than thyrotropin is more associated with metabolic parameters in healthy euthyroid adult subjects. Clin. Endocrinol..

[60] Yu T., Tian C., Song J., He D., Wu J., Wen Z., Sun Z., Sun Z. (2018). Value of the ft3/ft4 ratio and its combination with the grace risk score in predicting the prognosis in euthyroid patients with acute myocardial infarction undergoing percutaneous coronary intervention: A prospective cohort study. BMC Cardiovasc. Disord..

[61] da Silva L.E.S., Gouvêa E.C.D.P., Stopa S.R., Tierling V.L., Sardinha L.M.V., Macario E.M., Claro R.M. (2021). Data resource profile: Surveillance system of risk and protective factors for chronic diseases by telephone survey for adults in brazil (vigitel). Int. J. Epidemiol..

