# Supplementary material for: Branched-Chain Amino Acids, Alanine, and Thyroid Function: A Cross-Sectional, Nuclear Magnetic Resonance (NMR)-Based Approach from ELSA-Brasil
Source: Metabolites. 2024 Aug 6;14(8):437. doi: 10.3390/metabo14080437 (PMC11356049; doi:10.3390/metabo14080437)
Supplement: Supplementary file 1 [file metabolites-14-00437-s001.zip › metabolites-3035444-supplementary.pdf]

Supplementary table S1. Crude and adjusted linear association between thyroid parameters, subclinical hypothyroidism and amino acids according to sex.

	Valine		Leucine		Isoleucine		Alanine	
	**B (CI 95%)**	**p**	**B (CI 95%)**	**p**	**B (CI 95%)**	**p**	**B (CI 95%)**	**p**
**Male**								
**TSH**[a]	2.26 (-2.46;6.98)	0.349	3.08 (-0.57;6.73)	0.098	1.63 (-0.46;3.73)	0.127	16.19 (6.77;25.61)	0.001
**FT4**	-3.31 (-13.46;6.85)	0.523	-2.50 (-10.36;5.36)	0.533	-3.81 (-8.32;0.69)	0.097	-11.34 (-31.65;8.97)	0.274
**FT3**	27.78 (-1.54;57.10)	0.063	13.01 (-9.70;35.71)	0.262	-1.37 (-14.40;11.65)	0.836	34.49 (-24.20;93.18)	0.249
**FT3:FT4 ratio**	30.94 (0.49;61.39)	0.046	17.36 (-6.22;40.93)	0.149	7.60 (-5.92;21.12)	0.271	60.78 (-0.14;121.70)	0.051
**Subclinical hypo**[b]	2.84 (-2.95;8.63)	0.336	3.06 (-1.43;7.55)	0.181	2.37 (-0.21;4.96)	0.072	11.59 (-0.04;23.22)	0.051
**Female**								
**TSH**[a]	0.76 (-3.17;4.69)	0.703	0.82 (-2.11;3.74)	0.584	0.68 (-0.86;2.22)	0.390	12.78 (3.68;21.88)	0.006
**FT4**	-8.28 (-17.11;0.56)	0.066	0.46 (-6.13;7.05)	0.891	-3.87 (-7.33;-0.41)	0.028	-15.54 (-36.03;4.95)	0.137
**FT3**	39.35 (0.85;77.84)	0.045	30.11 (1.43;58.78)	0.040	7.26 (-7.85;22.36)	0.346	33.03 (-56.33;122.38)	0.469
**FT3:FT4 ratio**	53.75 (19.86;87.64)	0.002	18.89 (-6.40;44.19)	0.143	17.45 (4.15;30.75)	0.010	78.08 (-0.63;156.79)	0.052
**Subclinical hypo**[b]	0.30 (-4.60;5.21)	0.904	-1.12 (-4.78;2.54)	0.547	1.78 (-0.15;3.71)	0.070	8.44 (-2.89;19.78)	0.144

Note: [a]log-transformed variable. [b]Euthyroidism was the reference category. Analyses were adjusted for sociodemographic variables (age, race, educational level) and health conditions (BMI, physical activity, diabetes and smoking)

Supplementary table S2. Crude and adjusted linear association between thyroid parameters, subclinical hypothyroidism and amino acids according to age groups.

	Valine		Leucine		Isoleucine		Alanine	
	**B (CI 95%)**	**p**	**B (CI 95%)**	**p**	**B (CI 95%)**	**p**	**B (CI 95%)**	**p**
**Adults (<50 years-old)**								
**TSH[a]**	3.25 (-1.37;7.86)	0.168	3.06 (-0.47;6.60)	0.089	2.21 (0.23;4.19)	0.029	17.45 (7.25;27.65)	0.001
**FT4**	-10.76 (-20.62;-0.89)	0.033	-0.56 (-8.13;7.01)	0.884	-6.23 (-10.46;-2.00)	0.004	-17.18 (-39.05;4.70)	0.124
**FT3**	45.38 (1.21;89.56)	0.044	53.94 (20.17;87.70)	0.002	7.44 (-11.54;26.41)	0.442	4.38 (-93.61;102.38)	0.930
**FT3:FT4 ratio**	77.74 (38.11;117.38)	<0.001	43.89 (13.49;74.28)	0.005	30.32 (13.30;47.34)	<0.001	83.85 (-4.23;171.94)	0.062
**Subclinical hypo[b]**	1.70 (-4.08;7.48)	0.565	0.23 (-4.19;4.66)	0.917	1.32 (-1.16;3.81)	0.297	15.23 (2.48;27.98)	0.019
**Older adults (≥50 years-old)**								
**TSH[a]**	0.08 (-4.02;4.18)	0.969	1.12 (-2.00;4.24)	0.482	0.32 (-1.40;2.03)	0.718	13.11 (4.51;21.70)	0.003
**FT4**	-2.78 (-12.01;6.46)	0.555	-0.74 (-7.76;6.28)	0.837	-2.52 (-6.38;1.33)	0.199	-10.29 (-29.68;9.10)	0.298
**FT3**	26.88 (-0.22;53.99)	0.052	10.81 (-9.85;31.46)	0.305	-0.37 (-11.70;10.96)	0.949	35.52 (-21.44;92.47)	0.222
**FT3:FT4 ratio**	24.43 (-3.17;52.03)	0.083	11.82 (-9.20;32.85)	0.270	4.21 (-7.33;15.74)	0.475	52.97 (-4.99;110.94)	0.073
**Subclinical hypo[b]**	1.09 (-3.95;6.13)	0.671	1.07 (-2.78;4.92)	0.585	2.32 (0.20;4.44)	0.032	7.93 (-2.69;18.55)	0.144

Note: [a]log-transformed variables. [b]Euthyroidism was the reference category. Analyses were adjusted for sociodemographic variables (sex, race, educational level) and health conditions (BMI, physical activity, diabetes and smoking)

Supplementary table S3. Crude and adjusted linear association between thyroid parameters, subclinical hypothyroidism and amino acids according to the presence of diabetes.

	Valine		Leucine		Isoleucine		Alanine	
	**B (CI 95%)**	**p**	**B (CI 95%)**	**p**	**B (CI 95%)**	**p**	**B (CI 95%)**	**p**
**Absence of diabetes**								
**TSH[a]**	1.79 (-1.43;5.00)	0.276	2.33 (-0.16;4.82)	0.067	1.04 (-0.32;2.40)	0.134	15.91 (8.86;22.95)	<0.001
**FT4**	-11.47 (-18.62;-4.33)	0.002	-4.76 (-10.30;0.78)	0.092	-6.92 (-9.94;-3.90)	<0.001	-22.15 (-37.85;-6.45)	0.006
**FT3**	64.18 (31.35;97.01)	<0.001	50.23 (24.80;75.66)	<0.001	10.23 (-3.69;24.15)	0.150	31.29 (-40.98;103.55)	0.396
**FT3:FT4 ratio**	78.26 (50.02;106.51)	<0.001	46.82 (24.90;68.74)	<0.001	31.27 (19.31;43.23)	<0.001	98.80 (36.56;161.03)	0.002
**Subclinical hypo[b]**	1.57 (-2.36;5.50)	0.433	0.67 (-2.38;3.72)	0.669	1.59 (-0.08;3.26)	0.062	9.14 (0.50;17.77)	0.038
**Presence of diabetes**								
**TSH[a]**	-0.86 (-9.63;7.92)	0.848	0.31 (-6.03;6.65)	0.924	1.33 (-2.33;4.99)	0.476	7.48 (-9.69;24.65)	0.393
**FT4**	20.96 (2.56;39.37)	0.026	15.82 (2.52;29.11)	0.020	9.60 (1.93;17.28)	0.014	21.60 (-14.53;57.73)	0.241
**FT3**	11.75 (-24.54;48.05)	0.526	0.13 (-26.11;26.36)	0.992	-4.05 (-19.19;11.09)	0.600	43.13 (-27.88;114.15)	0.234
**FT3:FT4 ratio**	-9.26 (-50.58;32.05)	0.660	-15.10 (-44.94;14.74)	0.321	-14.58 (-31.79;2.62)	0.097	34.61 (-46.26;115.48)	0.402
**Subclinical hypo[b]**	1.45 (-10.09;13.00)	0.805	2.80 (-5.57;11.17)	0.512	4.36 (-0.48;9.20)	0.077	13.64 (-9.05;36.33)	0.239

Note: [a]log-transformed variables. [b]Euthyroidism was the reference category. Analyses were adjusted for sociodemographic variables (age, sex, race, educational level) and health conditions (BMI, physical activity, and smoking)

Supplementary table S4. Crude and adjusted linear association between thyroid parameters and amino acids for euthyroid participants exclusively.

	Valine		Leucine		Isoleucine		Alanine	
	**B (CI 95%)**	**p**	**B (CI 95%)**	**p**	**B (CI 95%)**	**p**	**B (CI 95%)**	**p**
**TSH[a]**	1.74 (-2.44;5.93)	0.415	3.63 (0.46;6.81)	0.025	0.28 (-1.50;2.05)	0.759	18.90 (9.98;27.82)	<0.001
**FT4**	-3.78 (-10.81;3.24)	0.291	0.19 (-5.14;5.52)	0.944	-3.02 (-5.99;-0.04)	0.047	-9.91 (-24.91;5.09)	0.195
**FT3**	32.46 (8.94;55.97)	0.007	17.49 (-0.37;35.34)	0.055	2.19 (-7.79;12.16)	0.668	36.00 (-14.25;86.26)	0.160
**FT3:FT4 ratio**	36.09 (12.82;59.37)	0.002	14.66 (-3.03;32.34)	0.104	9.91 (0.03;19.78)	0.049	60.89 (11.15;110.63)	0.016

Note: [a]log-transformed variables. Analyses were adjusted for sociodemographic variables (age, sex, race, educational level) and health conditions (BMI, physical activity, diabetes and smoking)

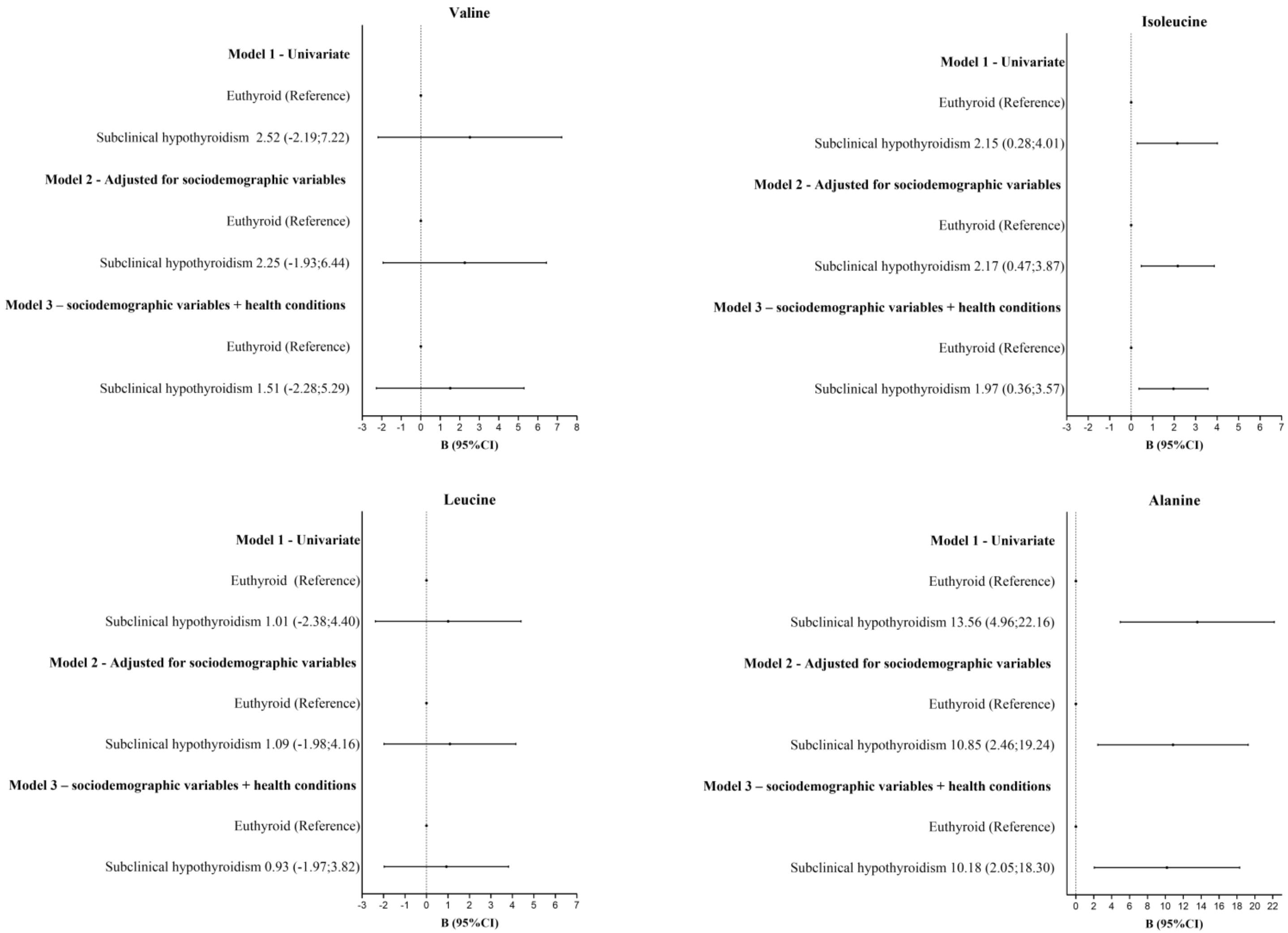

Supplementary Figure S1. Forest plots showing crude and adjusted linear association between subclinical hypothyroidism and amino acids. Note: B represents the slope coefficient of the regression line
